# Transporter Associated with Antigen Processing Proteins (TAP-1 and TAP-2) Gene Expression of MHC-I Downregulated in Oral Squamous Cell Carcinoma

**DOI:** 10.2174/0118715303344715241225184322

**Published:** 2025-02-11

**Authors:** Vijay Singh, Shailendra Dwivedi, Ruchika Agrawal, Mohanraj Palani Selvam, Akash Bansal, Akash Agarwal, Sanjeev Misra

**Affiliations:** 1 Department of Biochemistry, All India Institute of Medical Sciences Gorakhpur, 273008, India;; 2 Department of ENT, All India Institute of Medical Sciences Gorakhpur, 273008, India;; 3 Department of Surgical Oncology, Dr. Ram Manohar Lohia Institute of Medical Sciences Lucknow, 226010 India;; 4 Department of Onco-surgery, Atal Bihari Bajpayee Medical University Lucknow, 225001, India;; 5 Department of Onco-surgery, All India Institute of Medical Sciences, Jodhpur, 243005, India

**Keywords:** Oral squamous cell carcinoma, pre-malignant Oral Submucous Fibrosis (OSMF), Oral Lichen Planus (OLP), leukoplakia, TAP-1, TAP-2, MHC-1, inflammatory markers, C-reactive protein, creatinine, ferritin, ESR, gene expression

## Abstract

**Background:**

TAP-1 and TAP-2 are crucial proteins for loading antigenic peptides after proteasome-mediated endogenous processing of the MHC-I (Major Histocompatibility Complex-I) pathway. Our study aimed to explore the Transporter Associated with Antigen Processing proteins (TAP-1 and TAP-2) in oral squamous cell carcinoma and premalignant oral lesions.

**Methods:**

We recruited a total of 135 subjects from the outpatient department of the ENT unit of our institute. Real-time Polymerase Chain Reaction (PCR) was used to evaluate the levels of TAP-1 and TAP-2 gene expression in pre-cancerous and oral squamous carcinoma samples. Additionally, we measured the circulating levels of inflammatory markers using an automated biochemistry analyzer.

**Results:**

In the current study, we found that the subjects with oral squamous cell carcinoma had lower expressions of the TAP 1 and TAP 2 genes than precancerous oral subjects of OSMF, leukoplakia, and OLP. In oral squamous carcinoma subjects, we found a 1.7- and 2.1-fold change in gene expression of TAP-1 and TAP-2, respectively, compared to control subjects. Furthermore, we observed an increased levels of metabolic inflammatory biomarkers, including CRP, ESR, and ferritin in oral squamous carcinoma subjects compared to premalignant cases and controls, indicating the presence and aggravation of systemic inflammation.

**Conclusion:**

The study revealed that subjects with oral squamous cell carcinoma have lower TAP 1 and TAP 2 gene expression than premalignant control subjects, thus affecting MHC-I processing, which ultimately affects the functioning of the immune system. These results have the potential to improve our understanding of disease pathophysiology and provide more targeted treatment options.

## 1 INTRODUCTION

Oral squamous cell carcinoma is common in Southeast Asian countries, like India. According to a current WHO study, mouth cancer is rising globally and will rise up to 30% by 2030. About 1 million more cases of mouth cancer would arise annually [1, 2]. Oral Squamous Cell Carcinoma (OSCC) is a significant public health concern in India, where it constitutes one of the most prevalent forms of cancer, particularly among men. According to the Global Cancer Observatory (GLOBOCAN) 2020 data, India recorded approximately 135,929 new cases of lip and oral cavity cancer, with OSCC comprising the majority of these cases [3]. The incidence rate in India is approximately 10 per 100,000 individuals, with a notably high prevalence in areas where tobacco, betel nut, and areca nut products are widely consumed [4, 5]. Biomarker data in scientific databases is abundant, but only a biopsy can definitively diagnose all malignancies [6, 7]. Disturbances in homeostasis, caused by alterations in biochemical and physiological processes, result in the disease's manifestations. These alterations in physiochemical properties impact metabolic, immunological, and physiological processes [8, 9].

Over the past three decades, scientists have relentlessly explored the puzzle of carcinogenesis and found that inflammation and the immune system have a role in carcinogenesis and the aggravation of the disease [10, 11]. Cancer biology is also evolving, now encompassing cancer cells and the Tumor Microenvironment (TME), composed of inflammatory cells, fibroblasts, vascular cells, and the body's immune system [12, 13].

Modern cutting-edge technologies have led to advancements in single-cell omics, enabling exploration and modulation at single-cell levels, and potentially enhancing both understanding and management [14, 15]. With the advent of gene therapy and gene editing, it is now possible to control and intervene at cellular levels, revolutionizing the treatment of many diseases, including cancer management [16-19]. Following the drafting of the human genome, we have come to understand that optimal interactions of genes, nutrition, and environment are necessary for the proper functioning of physiochemical processes and immune biology [20-22]. The change in gene expression in immune molecules also affects the immune system's proper functioning. The proper processing and loading of antigens before their presentation to T cells is necessary for a positive immune response. MHC-I, which comprises many proteins, like TAP-1 and TAP-2 proteins that help with transport and chaperones, makes it possible for the body to process antigens on its own in the rough endoplasmic reticulum [23, 24].

The TAP-1 and TAP-2 proteins are important from the immune system's point of view. TAP-1 and TAP-2 are heterodimeric subunits that constitute a complex Transporter associated with Antigen Processing (TAP). They play a crucial role in transporting peptides into the endoplasmic reticulum, where the immune system receives them. This process is critical for identifying viral and tumor-derived peptides by immune system cells, such as CD8^+^ T-cells. Defects in TAP-1 and TAP-2 can lead to impaired antigen presentation and compromised immune function [25, 26].

Tumors can evade the immune response by interfering with the MHC class I antigen processing pathway. Tumors can employ selective mechanisms to modify the levels of TAP1 or TAP2 (or both) to reduce peptide transport (and their subsequent binding to MHC class I peptides). This ultimately affects the immune system's function, and the tumors may be able to evade detection by cytotoxic CD8^+^ T cells [27, 28].

In addition, inflammation, an important hallmark of all cancers, activates and affects innate and adaptive immune cells. Inflammation was initially known for its involvement in the host's immune response to infections, but now its role is evident in all types of cancer [12, 29]. Thus, we also plan to explore the clinically well-established inflammatory biomarkers in oral squamous carcinoma, pre-malignant subjects, and healthy controls. These inflammatory values may further aid in distinguishing premalignant conditions from cancers. Our study group hypothesized that dysregulated gene expression of TAP-1 and TAP-2, as well as inflammatory molecules, may be responsible for the development and progression of premalignant conditions and oral squamous cell carcinoma.

Researchers have investigated the expressions of TAP-1 and TAP-2 genes in various cancers, revealing varying expression patterns. For instance, breast cancer showed an increase in TAP-1 expression [30], whereas colorectal, renal cell, cervical, and melanomas showed a decrease [26]. We also observe discrepancies in the results of oral carcinomas, such as their upregulation [31] and downregulation [32]. Our study aimed to explore the gene expressions of TAP-1 and TAP-2 in subjects with oral squamous carcinoma, comparing them with premalignant subjects and healthy controls, as these findings are confused with actual gene expression.

## 2 MATERIALS AND METHODS

### 2.1 Patients’ Characteristics

This case-control research study was conducted after the ethical approval from the human ethics committee of the institute (IHEC/AIIMS-GKP/BMR/118/2023). The sample size was calculated with the assumption of 5.00% precision and an estimated prevalence of 2.70% based on previous research [33], assuming an infinite population size. These parameters facilitated the calculation of a 95% confidence interval, yielding bounds of 0% to 7.7%, reflecting the estimated prevalence adjusted by the specified precision. Although the initial sample size estimate indicated that 41 participants would provide statistical reliability, 45 samples were ultimately collected to enhance the robustness of the study and accommodate practical considerations.

Thus, the study recruited 135 subjects after they consented to participate, including 45 subjects with oral squamous cell carcinoma, 45 with premalignant lesions (leucoplakia, OSMF, and lichen planus), and 45 healthy normal individuals as controls. Patients who participated in the research were aged between 18 and 70 years, comprising both male and female individuals. Eligibility needed a confirmed diagnosis, either clinically or pathologically, ensuring that all participants had a documented disease relevant to the research. The study employed age and gender as criteria to compare premalignant and malignant cancer patients with normal healthy controls. Blood samples were analyzed, and participants were divided into clinical subgroups (pre-malignant lesions/malignant lesions) following confirmation of pathological (biopsy) approval. The research used the 8th edition of the American Joint Committee on Cancer (AJCC) standards for clinical and pathological staging. The exclusion criteria were those aged 70 years or older with no past history of cancer or inflammatory sickness, regardless of treatment.

### 2.2 Questionnaire and Data Processing

A questionnaire was used to collect information on biographical details, profession, medical treatment, food habits, family history, and lifestyle habits of individuals diagnosed with cancer. The questionnaire has been filled at the Ear, Nose, and Throat (ENT) outpatient department of AIIMS, Gorakhpur India. It included information on demographic, personal, habitual, nutritional, occupational, and medical aspects. The proforma also recorded medical history, including treatments, eating patterns, and work information. Cancer-related data were compiled, including the date of diagnosis, tumor size, grade, and degree of differentiation. This all-inclusive approach aimed to recognize the complex factors affecting cancer risk, disease progression, and therapy effectiveness, ultimately aiding in personalized treatment plans.

### 2.3 Laboratory Experimentation

This study involved the collection of a 3 ml intravenous blood sample, 400 µl collected in Trizol solution to isolate total RNA for analysing the Transporter Associated with Antigen Processing proteins (TAP-1 and TAP-2) gene expression. The remaining 2.6 ml was used to estimate inflammatory markers (CRP, creatinine, ferritin, and ESR) levels in serum or plasma; the sample was taken during the admission of cancer patients before treatment began.

#### 2.3.1 Genomic RNA Isolation

Peripheral blood has been used to isolate genomic RNA using Tri-Reagent and total RNA isolation kits (Qiagen and G-Biosciences). The quantification of RNA was done by measuring absorbance at 260 and 280 nm using a nanoplate reader (Biotek Instruments, USA). The integrity of the RNA was assessed *via* visual examination of the two ribosomal RNAs, 18S and 28S, on an agarose gel [34].

#### 2.3.2 Relative mRNA Quantification of Transporter Associated with Antigen Processing Proteins (TAP-1 and TAP-2) Gene

The relative quantification and fold expression of mRNA of the TAP genes were determined by extracting the total RNA from the blood cells. Complementary DNA was prepared from the whole RNA utilizing G Biosciences and Takara kits. The expression levels of genomic RNA were analyzed by employing gene quantification by Sybr green chemistry (2×AB HS SYBR Green qPCR mix: G Biosciences kit) on real-time PCR (Quant Studio 5) as per protocols. The primers of the housekeeping genes (GAPDH, beta-actin, and B-2 microglobulin) and target TAP-1 and TAP-2 genes (Eurofins Genomics LLC) were used for measuring the gene expression. The QuantStudio^TM^ Design and Analysis Software was used to analyze the results. According to the software, the data were created as sigmoid-shaped amplification plots in which the number of cycles was plotted against fluorescence. The crossing point (Cp/Ct/Cq) threshold point functions as a tool for the calculation of the starting template amount in each sample. The melting curves were assessed for each gene and the PCR reaction products were separated on a 2% agarose gel and stained with ethidium bromide to prove the presence of a single product. The efficiency of the action was assessed by amplifying serial dilutions of cDNA (1:10, 1:100, 1:1000, and 1:10,000). We confirmed the relationship between the threshold cycle and the log (RNA) to be linear (-3.6 < slope< 3.2). All experiments were performed in triplicate, and the relative amounts of target genes were normalized against the expression of the average of all three housekeeping genes for controls and case subjects.

#### 2.3.3 Serum Separation and Analysis of Metabolic Inflammatory Biomarkers

For serum separation, we used 2.6 ml of the 3 ml volume of blood and centrifuged it at a speed of 3000 revolutions per minute for 15 minutes at room temperature. We measured CRP, creatinine, ferritin, and ESR levels in the separated serum using estimation kits (Erba Mannheim; Mannheim, Germany) on a fully automated analyzer (XL 1000, from Transasia Bio-Medicals Ltd.). To analyze the parameters, we checked the instrument's external and internal quality control for proper and optimal functioning.

### 2.4 Calculations

#### 2.4.1 Relative and Fold Change Gene Expressions

Relative gene expressions of TAP 1 and TAP 2 of both oral squamous carcinoma patients and controls were calculated using [1/2] ΔCt, while fold change expressions were estimated using the equation 2-ΔΔCt [51] Thus, Ct values were normalized to the housekeeping gene, as ΔCt, which was determined by the formula ΔCt = Ct (target gene) -Ct (housekeeping gene). The TAP gene expression of healthy control subjects was used as a control to calculate the fold expression [35].

#### 2.4.2 Statistical Analysis

We compared TAP gene expressions and inflammatory biomarkers, such as CRP, ferritin, ESR, and creatinine levels, between and among various groups (premalignant, oral squamous carcinoma, and controls) using the independent t-test and ANOVA, respectively, based on the number of groups. For these analyses and comparisons, SPSS 27.0 and GraphPad Prism software version 8 were used, and *P* < 0.05 was considered statistically significant. The correlation between inflammatory biomarkers and TAP gene expression levels in subjects with oral squamous cell carcinoma was analyzed using Spearman correlation, employing IBM SPSS version 27.0 for the analysis Table **S1**.

## 3 RESULTS

### 3.1 Characteristic Parameters Distribution of Oral Squamous Carcinoma, Pre-Malignant Groups, and Controls

Various characteristics and risk factors were compared of the recruited subjects, as shown in Table **1**. The study emphasized on oral squamous cell carcinoma and its three degrees of differentiation: well-differentiated (G1), moderately differentiated (G2), and poorly differentiated (G3). It also evaluated premalignant oral conditions in detail, like Oral Lichen Planus (OLP), Oral Submucous Fibrosis (OSMF), and leukoplakia. The most common cases were males, within the age range of 45-70 years. The findings showed tobacco use in its various forms to be very common among all ages, with OSMF being more common in younger males aged 25-55 years. Leukoplakia has been a frequent oral problem among adults in their 30s and 40s consuming significant amounts of tobacco.

### 3.2 Transporter Associated with Antigen Processing Proteins (TAP-1 and TAP-2) Gene Expressions (Relative Gene and Fold Change) in Pre-Malignant Subjects

The TAP-1 gene expression had been upregulated in premalignant oral subjects, with a 4.46-fold increase in oral sub-mucous fibrosis (*p* < 0.0001), a 2.16-fold increase in leukoplakia, but it was non-significant (*p* = 0.0567), and a 4.18-fold change in Oral Lichen Planus (OLP) (*p* < 0.01) in comparison to healthy controls.

When compared to healthy controls, the expression of the TAP-2 gene was slightly upregulated in oral premalignant subjects, with fold changes of 0.91 in OSMF (*p* = 0.20) and 1.4 fold increase in leukoplakia (*p* < 0.90). However, it had been significantly downregulated in the case of OLP, with a fold change of 3.89 (*p* < 0.0001). Overall, TAP 2 gene expression was 3.89-fold (*p* < 0.01) lower in OLP and 1.4-fold higher (*p* < 0.05) in leukoplakia compared to controls. However, there had been a decrease in the value of the TAP 2 gene expression in the subjects of OSMF (*p* = 0.78), as compared to leukoplakia. The overall comparison of gene expression (relative gene and fold change) of TAP-1 and TAP-2 proteins of pre-malignant and controls has been summarised in Table **2**.

### 3.3 Transporter Associated with Antigen Processing Proteins (TAP-1 and TAP-2) Gene Expressions in Oral Squamous Cell Carcinoma Subjects

The findings showed a decrease in TAP-1 and TAP-2 gene expressions in oral squamous cell carcinoma compared to the control group (*P* < 0.01). The study revealed a down-regulation of TAP-1 and TAP-2 gene expressions by 1.71 and 2.18 times, respectively, in comparison to the control subjects. A similar trend was also noticed in the relative gene expressions of both genes. Table **3** summarizes the comparison of TAP expression patterns (TAP 1 and TAP 2) (relative gene and fold change) in oral squamous cell carcinoma.

### 3.4 Transporter Associated with Antigen Processing Proteins (TAP-1 and TAP-2) Gene Expressions in subjects of Oral Squamous Cell Carcinoma Subjects and Premalignant Oral Lesions

Table **4** presents a comparison of gene expression between premalignant subjects and those with oral squamous cell carcinoma, in terms of the fold change and relative gene expression. We standardized the expressions of the TAP-1 and TAP-2 genes by comparing them to the expressions of housekeeping genes. We analysed the expressions of TAP-1 and TAP-2 genes in two groups of participants: one consisting of persons diagnosed with oral squamous cell carcinoma, and the other consisting of individuals with pre-malignant cells, specifically OLP, OSMF, and leukoplakia. The expressions of TAP-1 genes in the oral squamous cell carcinoma group were noticeably decreased (*P* < 0.01) compared to the group of individuals with pre-malignant oral lesions. For individuals with premalignant oral conditions, the TAP 1 gene exhibited a fold change of 3.79, significantly more than the fold change of 1.706 observed in the TAP 1 gene of oral squamous cells. The key factor contributing to the observed disparity was the higher magnitude of change in gene expression in OSMF participants compared to oral squamous cell carcinoma subjects, as can be seen in Table **2**. In addition, the fold change in TAP-2 gene expression exhibited a smaller difference in both groups (2.58 *vs.* 2.18), but it was still statistically significant.

### 3.5 Metabolism-Associated Inflammatory Biomarkers Levels Among Premalignant, Oral Squamous Cell Carcinoma, and Control Subjects

The study examined the endogenous inflammatory markers of ESR (Erythrocyte Sedimentation Rate), creatinine, ferritin, and CRP (C-reactive Protein) among all three groups: control, pre-malignant, and oral squamous cell cancer. When comparing ESR levels across all groups tested, there was a substantial increase (*p* < 0.05) noticed in subjects of OSMF in the pre-malignant group, as well as in cases of oral squamous cell carcinoma. An ascending trend of ESR was observed in subjects with leukoplakia, Oral Lichen Planus (OLP), Oral Submucous Fibrosis (OSMF), and Oral Squamous Cell Carcinoma (OSCC). However, the rise in value was not statistically significant for leukoplakia and OLP. In addition, the OSMF group exhibited a substantial increase (*p* < 0.01) in serum creatinine levels compared to the control group. Moreover, the ferritin levels were found to be significantly increased (*p* < 0.01) in instances of oral squamous cell carcinoma compared to the control group. Furthermore, the levels of CRP (C-reactive Protein) were elevated in individuals with leukoplakia of the pre-malignant group. Table **5** provides a summary of the circulatory levels of all metabolically related inflammatory indicators.

### 3.6 Correlation Between Inflammatory Markers and TAP Protein Expression in Oral Squamous Cell Carcinoma

In oral squamous cell carcinoma, elevated inflammatory marker levels exhibited diverse correlations with TAP1 and TAP2 expression, potentially indicating mechanisms of immune evasion. ESR exhibited a weak positive correlation with TAP1 (r = 0.1856), suggesting a possible inflammatory influence on TAP1-mediated immune surveillance. Ferritin exhibited a moderate negative correlation with TAP1 (r = -0.1643) and TAP2 (r = -0.0667), potentially indicating iron sequestration to facilitate tumor growth while diminishing TAP expression. CRP exhibited weak negative correlations with TAP1 (r = -0.0668) and a weak positive correlation with TAP2 (r = 0.1720), indicating a complex role in inflammatory immune processing. Finally, creatinine exhibited weak positive (TAP1, r = 0.0347) and negative (TAP2, r = -0.1852) correlations, suggesting the influence of broader inflammation on immune evasion. These correlations indicated a potential relationship between inflammation and diminished antigen presentation in the progression of OSCC. Table **6** illustrates the strength and direction of correlations between each inflammatory marker and TAP1/TAP2 expression levels in OSCC patients. Positive values signify a direct correlation, whereas negative values denote an inverse correlation.

## 4 DISCUSSION

This is the first study to evaluate and compare the Transporter Associated with Antigen Processing proteins (TAP-1 and TAP-2) gene expressions in oral premalignant and oral squamous cell carcinoma groups. The study findings of premalignant cases demonstrated the TAP-1 gene to be significantly upregulated in OSMF cases with a 4.46-fold change; this prominent upregulation indicated that TAP-1 might have a significant role in the development of this condition. TAP-1 is essential for the transport of antigenic peptides into the endoplasmic reticulum, which is needed for immune surveillance and the presentation of endogenous antigens to T cells. The marked increase could indicate an elevated immune response or an attempt by the cells to counter the fibrotic state [36].

Further, the study also observed a significant 4.18-fold upregulation of TAP-1 expression in OLP, suggesting an aggravated immune response. Since Oral Lichen Planus (OLP) is viewed as an autoimmune condition [37], the augmented TAP-1 expression could reveal the chronic inflammatory state where more antigen presentation persists over time to cope with the homeostasis of the micro-environment [38].

A 1.4-fold increase in TAP-2 gene expression (*p* < 0.90) was not statistically significant. This implies that, as with TAP-1, TAP-2 may not be a key player in leukoplakia development, and the study also suggested its variation across various disease conditions; this inconsistency in expression may be due to changes in its regulatory molecules, like interleukins, transcription factors, *etc* [38].

The TAP-1 gene was downregulated 1.71-fold in Oral Squamous Cell Carcinoma (OSCC) compared to control subjects; this downregulation of TAP-1 proposed a compromised antigen processing capability, leading to reduced presentation of tumor antigens on the cell surface. This could further weaken the immune system's ability to detect and eliminate cancerous cells, facilitating tumor immune evasion [39, 40].

The downgraded expression of TAP-1 in cases of OSCC was found to be inconsistent with the similar findings reported in bladder cancer [41, 42], small cell lung cancer [43, 44], glioma [45], prostatic cancer [46], Head and Neck Squamous Cell Carcinoma (HNSCC) [47], and breast cancer [30]. Although a study conducted on uveal melanoma showed elevated expression of TAP1 gene expression, this may be due to different pathogenesis of uveal melanoma development and progression [48].

Additionally, the TAP-2 gene was downregulated by 2.18-fold in OSCC compared to control subjects. Similar to TAP-1, the decreased expression of TAP-2 further suggested an immune escape mechanism affecting antigen processing and presentation. The downregulation of both TAP-1 and TAP-2 could have a synergistic negative effect on the overall efficiency of the MHC class I pathway, contributing to an escape from immune surveillance [49, 50]. Thus, with the decrease in the presentation of endogenous peptides, tumor cells can prevent recognition by cytotoxic T cells, allowing them to unrestricted proliferation. As per the updated mechanism, this downgraded expression might be due to mutations, deletions, or epigenetic silencing (*e.g.*, DNA methylation, histone modifications) of TAP genes that may alter their gene expression [39, 51].

Additionally, it is also established that molecules and factors within the tumor microenvironment, including cytokines and growth factors, could impact the expression of TAP genes. For example, Transforming Growth Factor-beta (TGF-β) and other immunosuppressive molecules are known to downregulate components of the antigen-processing machinery [52, 53]. The study examined the expressions of TAP-1 and TAP-2 in premalignant and oral squamous cell carcinoma; however, it is crucial to acknowledge that these proteins are merely one component of the numerous pathways implicated in the transition from premalignant conditions to Oral Squamous Cell Carcinoma (OSCC). Additional molecular mechanisms, such as further elements of the antigen presentation pathway and more extensive immune-modulatory pathways, are likely to affect disease progression and immune evasion. Future research should encompass wider analyses to achieve a more thorough understanding of these mechanisms.

The noted downregulation of TAP-1 and TAP-2 in OSCC corresponded with observations in several other malignancies, substantiating the idea that decreased TAP expression is a common approach by which tumors escape immune recognition [50]. In cervical cancer, downregulation of TAP-1 and TAP-2 has been noted, corresponding with worse patient prognosis and heightened tumor aggressiveness. The diminished TAP expression in cervical cancer may hinder the recognition of tumor cells by cytotoxic T lymphocytes, facilitating ongoing tumor proliferation despite an immune-active microenvironment [50]. In prostate cancer, decreased TAP expression is often correlated with advanced stages of the illness. Research indicates that when prostate cancer advances, it often develops methods to lower TAP levels, thereby reducing MHC-I presentation and evading immune surveillance [54, 55].

The downregulation of TAP for immune evasion is crucial in prostate cancer, since it may lead to resistance to immunotherapies dependent on T-cell-mediated responses [56]. In colorectal cancer, the downregulation of TAP proteins is associated with increased immune evasion and metastasis. Colorectal cancers characterized by diminished TAP expression often display decreased infiltration of cytotoxic T lymphocytes, essential for regulating tumor dissemination [57]. The results indicated that decreased TAP expression in colorectal cancer not only promotes immune evasion, but also likely enhances the tumor's spreading potential.

Downregulation of TAP-1 and TAP-2 has also been observed in breast cancer, especially in Triple-negative Breast Cancer (TNBC), a subtype characterized by its aggressive behavior and inadequate response to standard treatments. Downregulation of TAP in TNBC is thought to diminish antigen presentation, thus impairing T-cell-mediated immune responses and fostering resistance to checkpoint inhibitors [50, 58].

The current article also evaluated four metabolically associated inflammatory molecules, the first one being ESR, which is a non-specific marker of inflammation that upsurges with raised levels of fibrinogen and other acute-phase proteins in the blood. The considerable increase in ESR in subjects with OSMF and OSCC indicated an amplified inflammatory state. Chronic inflammation is also an important hallmark of cancers [59-62].

Thus, in subjects with OSMF, the significant rise suggests ongoing fibrotic and inflammatory processes. In OSCC, it reflects the systemic inflammatory response to malignancy. A non-significant increase in leukoplakia and OLP might indicate minor levels of systemic inflammation or varying inflammatory responses [63].

The second endogenous biomolecule chosen was creatinine, which is a by-product of muscle metabolism that is excreted via the kidneys. Raised serum creatinine levels in OSMF subjects could designate renal involvement or reduced renal function, which may be secondary to systemic inflammation, fibrosis, or other systemic effects of chronic disease. The chronic inflammatory state in OSMF could potentially affect the kidneys [64, 65].

Ferritin, another biomolecule examined in this study, is an acute-phase protein and an indicator of iron storage. Enhanced ferritin levels in OSCC cases revealed a prominent inflammatory and probably neoplastic activity, as cancer cells can sequester iron to facilitate their growth. High ferritin levels might also indicate increased iron storage secondary to chronic inflammation or the body's response to malignancy [66, 67].

CRP (C-reactive Protein) is also a sensitive marker of systemic inflammation, and its increased levels are seen in leukoplakia that exhibit an inflammatory micro-environment underlying this pre-malignant condition [68]. Albeit CRP is raised, it is more localized or specific compared to ESR, often representing an acute inflammatory response. The elevation of CRP in leukoplakia indicates a state of chronic irritation or mild inflammation, which could predispose to malignant transformation if left unchecked [69].

The consistent boost of these inflammatory markers across diverse stages from pre-malignant conditions (like OSMF, leukoplakia, OLP) to malignant states (OSCC) emphasizes the role of inflammation in the pathogenesis and progression of oral diseases. Chronic inflammation can contribute to DNA damage, and promote cancer cell survival, proliferation, angiogenesis, and metastasis [70, 71].

Considering our findings of TAP-1 and TAP-2 downregulation in OSCC, we propose future research directions that could deepen understanding and enhance treatment options. Mechanistic studies should investigate the genetic and epigenetic causes of TAP suppression in OSCC, which can potentially reveal new therapeutic targets [72]. Additionally, experiments to upregulate TAP proteins could assess whether restoring TAP expression enhances MHC-I antigen presentation and improves immune recognition [73]. Further exploration of other antigen-processing components, such as LMP2, LMP7, and ERAAP, and their interactions with TAP may provide a more comprehensive view of immune surveillance in OSCC [74, 75]. Finally, studies combining TAP modulation with immune checkpoint inhibitors in animal models could reveal synergistic effects, potentially improving immunotherapy outcomes for OSCC patients [76]. These investigations may pave the way for biomarker-driven immunotherapies that restore immune surveillance in OSCC.

## LIMITATIONS

A limitation of this study is that it was conducted based on the calculated sample size. For better predictive accuracy and generalizability, future studies with larger sample sizes will be necessary.

## CONCLUSION

In conclusion, the findings of TAP-1 and TAP-2 gene expression, along with metabolic inflammatory markers, underscore their potential as valuable biomarkers for early detection, prognosis, and disease monitoring in Oral Squamous Cell Carcinoma (OSCC) and pre-malignant conditions. The downregulation of TAP genes highlights a mechanism by which OSCC cells evade immune detection, suggesting that TAP expression levels could inform personalized treatment approaches, including the selection and enhancement of immunotherapy. Additionally, elevated levels of ESR, creatinine, ferritin, and CRP indicate underlying inflammatory and metabolic disruptions associated with OSCC progression. These biomarkers may not only aid in identifying high-risk patients, but also serve as targets for adjunct therapies that could enhance immune response and improve patient outcomes. Overall, this study paves the way for integrating TAP and inflammatory markers into OSCC management, which can help foster more tailored, biomarker-driven therapeutic strategies in clinical practice.

## Figures and Tables

**Table 1:** Characteristic parameters of oral squamous cell carcinoma, premalignant oral subjects, and control.

Characteristics	Oral Squamous Cell Carcinoma				Premalignant Oral Subjects					Control Subjects (45)
	Well-Differentiated (12)	Moderately Differentiated (23)		Poorly Differentiated (10)	OLP (22)	OSMF (11)			Leukoplakia (12)	
**Gender**										-
**Male**	8	19		6	13	10			9	29
**Female**	4	4		4	9	1			3	16
**Age (in years)**										
**20-30**	0	2	0		5		2	2		18
**30-40**	1	2	1		2		3	6		16
**40-50**	2	9	2		5		3	2		9
**50-60**	4	4	2		6		2	2		2
**60-70**	4	3	3		2		0	0		0
**70-80**	1	3	2		2		1	0		0
**Risk factor exposure**										
**Paan masala**	0	0	1		2		0	0		2
**Tobacco**	4	7	2		7		5	4		0
**Bidi**	1	2	1		0		0	0		1
**Alcohol**	1	2	1		1		0	2		0
**Tobacco and Alcohol**	4	8	1		2		4	2		0
**Paan masala and Alcohol**	0	1	1		1		0	1		1
**Tobacco, Alcohol, and cigarettes**	2	1	0		0		0	2		0
**Gutka and Alcohol**	0	1	1		1		0	0		0
**No Exposure**	**0**	**1**	**2**		**8**		**1**	**1**		**40**

**Note:** Statistical analysis in all comparisons have *p* > 0.05.

**Table 2:** Expression pattern of Transporter Associated with Antigen Processing Proteins (TAP-1 and TAP-2) and their gene (relative gene & fold change) expressions in pre-malignant subjects.

Study Groups	Gene TAP 1 TAP 2			Relative Gene Expression		Fold Change Gene Expression	
				TAP1 (1/2^∆CT^)	TAP2 (1/2^∆CT^)	TAP 1 (2^-∆∆CT^)	TAP 2 (2^-∆∆CT^)
**Control**	(Ct mean±SD)	24.77 ± 1.04	22.87 ± 0.94	Reference			
	(∆Ct mean±SD)	-7.20 ± 0.421	-5.30±0.33				
**OSMF**	(Ct mean±SD)	22.74 ± 1.61**	22.14 ±0.26*	0.024	0.031	4.46	0.911
	(∆Ct mean±SD)	-5.54±1.4 **	-4.94±0.057*				
**OLP**	(Ct mean±SD)	23.63 ± 2.06**	25.62 ± 0.68**	0.030	0.006	4.18	3.89
	(∆Ct mean±SD)	-5.30 ±1.71**	-7.29 ± 0.33**				
**Leukoplakia**	(Ct mean±SD)	23.32 ±0.19	21.9035±0.71*	0.012	0.033	2.16	1.4
	(∆Ct mean±SD)	-6.581± 0.10	-5.167± 0.624*				

**Note:** *P* ≤ 0.05 denoted by * and *p* <0.01 denoted by **.

**Table 3:** Expression pattern of Transporter Associated with Antigen Processing Proteins (TAP-1 and TAP-2) and their gene (relative gene & fold change) expressions in Oral squamous cell carcinoma subjects.

Study Groups	Gene TAP 1 TAP 2			Relative Gene Expression		Fold Change Gene Expression	
				TAP1 (1/2^∆CT^)	TAP2 (1/2^∆CT^)	TAP 1 (2^-∆∆CT^)	TAP 2 (2^-∆∆CT^)
**Control**	(Ct mean±SD)	24.77 ± 1.04	22.87 ± 0.946	Reference			
	(∆Ct mean±SD)	-7.20 ± 0.421	-5.30±0.33				
**Oral Squamous cell carcinoma**	(Ct mean±SD)	25.71±0.28^**^	25.21±0.078^**^	0.0107	0.0144	1.706	2.18
	(∆Ct mean±SD)	-6.72±1.34^**^	-6.22±1.13^**^				

**Note:** *P* ≤ 0.05 denoted by * and *p* <0.01 denoted by **.

**Table 4:** Expression pattern of Transporter Associated with Antigen Processing Proteins (TAP-1 and TAP-2) and their gene (relative gene & fold change) expressions in Oral squamous cell carcinoma subjects and Premalignant oral lesion.

Study Group	Gene TAP 1 TAP2			Relative Gene Expression		Fold Change Gene Expression	
				TAP1 (1/2^∆CT^)	TAP2 (1/2^∆CT^)	TAP 1 (2^-∆∆CT^)	TAP 2 (2^-∆∆CT^)
**Premalignant**	(Ct mean±SD)	23.33 ±1.36	23.90±2.73	0.025	0.019	3.79	2.58
	(∆Ct mean±SD)	-5.65 ± 0.78	-6.22 ± 1.54				
**Oral squamous cell carcinoma**	(Ct mean±SD)	25.71±0.28^**^	25.21±0.078^**^	0.0107	0.0144	1.706	2.18
	(∆Ct mean±SD)	-6.72±1.34^**^	-6.22±1.13^**^				

**Note:** *P* ≤ 0.05 denoted by * and *p* <0.01 denoted by **.

**Table 5:** Comparison of levels of inflammatory biomarkers such as (ESR, Creatinine, Ferritin, and CRP) among Premalignant, Oral squamous cell carcinoma and control subjects.

Condition		ESR	Creatinine	Ferritin	CRP
**Control** **(Reference)**		11.11 ± 5.42	0.73 ± 0.09	97.20 ± 41.56	5.33 ± 0.46
**Premalignant lesions**	Leukoplakia	13.33 ± 3.53^NS^ (*P* ≤ 0.245)	0.75 ± 0.05^NS^ (*P* ≤ 0.431)	91.54 ± 22.6^NS^ (*P* ≤ 0.695)	18.76±22.78** (*P* ≤ 0.01)
	OLP	16.59 ± 8.22* (*P* ≤ 0.002)	0.74 ± 0.144^NS^ (*P* ≤ 0.830)	110.24 ± 83.9^NS^ (*P* ≤ 0.396)	7.51 ± 2.755^*^ (*P* ≤ 0.01)
	OSMF	25.66±21.072** (*P* ≤ 0.01)	1.07 ± 0.49** (*P* ≤ 0.157)	122.62± 86.015^NS^ (*P* ≤ 0.157)	6.08 ± 0.54^NS^ (*P* ≤ 0.01)
**Oral Cancer**		26.28± 20.286^**^ (*P* ≤ 0.01)	0.82 ± 0.177 (*P* ≤ 0.03)	162.88± 115.49^**^ (*P* ≤ 0.001)	8.00±4.16** (*P* ≤ 0.001)

**Note:** *P* ≤ 0.05 denoted by * and *p* <0.01 denoted by **.

**Table 6:** Correlation between inflammatory biomarkers (ESR,CRP,Creatinine and Ferritin) and TAP gene expression in Oral Squamous cell carcinoma subjects.

Inflammatory Marker	TAP1 (R value)	TAP2 (R value)
**ESR**	0.1856	-0.0608
**Ferritin**	-0.1643	-0.0667
**CRP**	-0.0668	0.1720
**Creatinine**	0.0347	-0.1852

**Note:** Positive values indicate a direct correlation, while negative values indicate an inverse correlation.

## Data Availability

The data and supportive information are available within the article.

## LIST OF ABBREVIATIONS

TAP 1/2: Transporter Associated with Antigen Processing Proteins
OLP: Oral Lichen Planus
OSMF: Oral Submucous Fibrosis
ESR: Erythrocytic Sedimentation Rate
CRP: C-Reactive Protein
OSCC: Oral Squamous Cell Carcinoma
PCR: Polymerase Chain Reaction

## STANDARD FOR REPORTING

STROBE and SAGER guidelines were followed in this study.
