# Supplementary material for: Transporter Associated with Antigen Processing Proteins (TAP-1 and TAP-2) Gene Expression of MHC-I Downregulated in Oral Squamous Cell Carcinoma
Source: Endocr Metab Immune Disord Drug Targets. 2025 Feb 11;26:E18715303344715. doi: 10.2174/0118715303344715241225184322 (PMC13284669; doi:10.2174/0118715303344715241225184322)
Supplement: Supplementary file 1 [file EMIDDT-26-E18715303344715_SD1.pdf]

Supplementary Material

Transporter Associated with Antigen Processing Proteins (TAP-1 and TAP-2) Gene Expression of MHC-I Downregulated in Oral Squamous Cell Carcinoma

Vijay Singh[1, #], Shailendra Dwivedi[1, *, #], Ruchika Agrawal[2], Mohan Raj PS[1], Akash Bansal[1],

Akash Agarwal[3] and Sanjeev Misra[4]

[1]Department of Biochemistry, All India Institute of Medical Sciences Gorakhpur, 273008, India; [2]Department of ENT, All India Institute of Medical Sciences Gorakhpur, 273008, India; [3]Department of Surgical Oncology, Dr. Ram Manohar Lohia Institute of Medical Sciences Lucknow, 226010 India; [4]Atal Bihari Bajpayee Medical University, Lucknow, 225001, India; and All India Institute of Medical Sciences Jodhpur, 243005, India

Table S1: Primer sequence (housekeeping gene) and target gene**.**

**S.N.**	**Gene (Gene ID)**	**Forward Primer Sequences**	**Reversed Primer Sequences**
1.	GAPDH	GTCTCCTCTGACTTCAACAGCG	ACCACCCTGTTGCTGTAGCCAA
2.	BETA-ACTIN	CACCATTGGCAATGAGCGGTTC	AGGTCTTTGCGGATGTCCACGT
3	Beta-2 MICROGLOBULIN	CCACTGAAAAAGATGAGTATGCCT	CCAATCCAAATGCGGCATCTTCA
4	**TAP-1**	TGCCCCGCATATTCTCCCT	CACCTGCGTTTTCGCTCTTG
5	**TAP-2**	TGGACGCGGCTTTACTGTG	GCAGCCCTCTTAGCTTTAGCA
